# Telomere Attrition Due to Infection

**DOI:** 10.1371/journal.pone.0002143

**Published:** 2008-05-14

**Authors:** Petteri Ilmonen, Alexander Kotrschal, Dustin J. Penn

**Affiliations:** 1 Konrad Lorenz Institute for Ethology, Austrian Academy of Sciences, Vienna, Austria; Karolinska Institutet, Sweden

## Abstract

**Background:**

Telomeres–the terminal caps of chromosomes–become shorter as individuals age, and there is much interest in determining what causes telomere attrition since this process may play a role in biological aging. The leading hypothesis is that telomere attrition is due to inflammation, exposure to infectious agents, and other types of oxidative stress, which damage telomeres and impair their repair mechanisms. Several lines of evidence support this hypothesis, including observational findings that people exposed to infectious diseases have shorter telomeres. Experimental tests are still needed, however, to distinguish whether infectious diseases actually cause telomere attrition or whether telomere attrition increases susceptibility to infection. Experiments are also needed to determine whether telomere erosion reduces longevity.

**Methodology/Principal Findings:**

We experimentally tested whether repeated exposure to an infectious agent, *Salmonella enterica*, causes telomere attrition in wild-derived house mice (*Mus musculus musculus)*. We repeatedly infected mice with a genetically diverse cocktail of five different *S. enterica* strains over seven months, and compared changes in telomere length with sham-infected sibling controls. We measured changes in telomere length of white blood cells (WBC) after five infections using a real-time PCR method. Our results show that repeated *Salmonella* infections cause telomere attrition in WBCs, and particularly for males, which appeared less disease resistant than females. Interestingly, we also found that individuals having long WBC telomeres at early age were relatively disease resistant during later life. Finally, we found evidence that more rapid telomere attrition increases mortality risk, although this trend was not significant.

**Conclusions/Significance:**

Our results indicate that infectious diseases can cause telomere attrition, and support the idea that telomere length could provide a molecular biomarker for assessing exposure and ability to cope with infectious diseases.

## Introduction

Biological aging or senescence may be due to accumulating DNA damage in somatic cells [1], [2], including erosion of telomeres, which may be caused by exposure to infectious and inflammatory diseases [3], [4] and other forms of oxidative stress [5], [6]. Telomeres help to stabilize the genome by protecting chromosomes from end-to-end fusions and degradation during mitotic division [7], [8]. Because DNA only replicates from the 5′ to 3′ direction, telomeres become shorter with each cell division, and after a critical threshold has been reached, cells can no longer proliferate and arrest to senescence. The limit of cellular proliferative lifespan due to telomere shortening is suspected to be an important determinant of organismal senescence and life span [9]–[11]. Telomeres can be repaired by a special reverse transcriptase (telomerase), but *in vitro* studies show that telomeres are vulnerable to oxidative damage by reactive oxygen species (ROS) and that oxidative stress impairs the function of telomerase and other mechanisms that repair telomeres [12]. Thus, telomere shortening may be a *cause* of senescence, a *consequence* of oxidative stress or other mechanisms that cause aging, or both. Gradual telomere attrition appears to be a normal part of aging, whereas accelerated telomere loss and premature senescence is suspected to occur in particular in cells that are exposed to internal or external stressors, which provoke increased cellular proliferation and ROS production. Immune cells are expected to be particularly vulnerable to telomere shortening and accelerated senescence because of their rapid proliferation and high ROS production by phagocytes during inflammatory responses [3], [9]. The aim of our study was to test whether repeated exposure to an infectious agent, known to cause inflammation and oxidative stress, results in telomere attrition, and whether more rapid telomere attrition increases mortality.

Several lines of evidence suggest that infectious diseases cause telomere attrition, and that such changes could subsequently reduce immunocompetence and lifespan. First, observational studies in humans find that patients with chronic viral infections have short telomeres in specific T lymphocytes [3], [4], and patients with chronic inflammatory liver diseases show reduction in telomeres of hepatocytes [13]–[15]. Furthermore, for people older than 60 years, individuals with shorter telomeres in WBCs have increased mortality rates, attributable to infectious diseases [16], but see [17]. Similarly, people with a rare genetic disease causing telomerase-deficiency die prematurely due to vulnerability to infectious diseases [18]. Second, *in vitro* experiments show that telomerase activity in T lymphocytes initially increases with acute antigen exposure, but then drastically decreases with repeated encounters with the same antigenic stimulus [19]. Third, studies on telomerase-deficient mutant mice report that after the fourth generation these mice have shorter telomeres and a number of impairments, including reduced viability, decreased proliferative capacity of B and T lymphocytes and various diseases [20]. Also, experimental studies on telomerase-deficient mutant mice show that antigen stimulation results in accelerated telomere loss in splenocytes and consequent impairment of immune function [21], and telomere dysfunction is associated with defects in organ regeneration after experimentally induced liver cirrhosis [22].

Taken together, these findings support the idea that chronic exposure to infectious diseases can cause telomere shortening, immunosenescence, and reduced longevity; however, the critical experimental tests are lacking. Having short telomeres is associated with increased exposure to infectious and inflammatory diseases [3], [4], [13]–[15], but experiments are still needed to determine whether telomere attrition is caused by infectious agents or whether telomere length influences susceptibility to infection (and also to rule out the possibility that attrition is due to social stress or some other factor correlated with exposure to infectious agents). Also, the results from *in vitro* studies and from studies with telomerase-deficient mutant mice need to be verified with genetically intact hosts. Therefore, our approach was to test whether experimental exposure to actual infectious agents causes telomere attrition using wild house mice. Our results provide support for the idea that long-term exposure to pathogenic infections results in telomere attrition of WBCs, and also that telomere length of WBCs at early age may influence individuals' ability to cope with inflammatory infections later in life.

## Results and Discussion

We infected wild-derived house mice (*Mus musculus musculus)* every four weeks with a mixture of five different strains of *Salmonella enterica* (serovar Typhimurium and serovar Enteritidis) over seven months, and compared changes in their telomeres to sham-infected controls. Once ingested, *Salmonella* passes the intestinal epithelium, and then proliferates in high concentrations within macrophages and dendritic cells of the spleen and liver. Infection results in massive expansion of these tissues, and triggers strong responses from both innate (high ROS production) and acquired (high lymphocyte proliferation) arms of the immune system [23], [24]. We used wild mice because despite the importance of laboratory strains as model organisms they also have several potential problems when used in aging research. First, laboratory strains are often heavily inbred, which has led to loss of alleles due to founder effects and purging [25]. Second, during domestication inbred strains have been selected for early and high reproductive output, which may have resulted to loss of alleles that retard the aging process [26]. Third, laboratory strains have unusually long telomeres compared to their wild counterparts, raising concerns about the relevance of results to humans and wild mammals [27]. We measured the change in WBC telomere length after five infections in WBCs and the relative telomere lengths (T/S ratio) in hepatocytes and splenocytes at termination with a real-time PCR method [28] and compared experimentally infected mice to sham-infected siblings.

At the termination of the experiment, 86 % of infected males (6/7) and 50 % of females (5/10) were still infected with *Salmonella*. Experimentally infected males and females showed enlarged spleens and livers compared to sham-controls, but no statistically significant differences in body weights (Table 1.). Four treatment males (36 %) and two females (17 %) died during the experiment. Among infected mice, males also showed higher *Salmonella* prevalence, bacterial loads and more evidence of pathology (enlarged spleens and livers) than their sisters, although these differences were significant only for liver mass (Table 2.). These results indicate that the repeated experimental inoculation resulted in systemic infection and disease pathology, and that males were somewhat more susceptible than females.

**Table 1:** Pair-wise sib-sib comparisons for experimentally *Salmonella*-infected versus sham-treated mice for spleen and liver mass, body mass prior to the first infection, prior to the sixth infection and at termination of the experiment.

	*Salmonella*-infected		Sham-infected			
	N	mean±s.e.m.	N	mean±s.e.m.	test	P-value
**Males**						
Spleen mass	7	0.14±0.05	7	0.05±0.00	Z = −1.99	**0.02**
Liver mass	7	1.47±0.14	7	1.15±0.06	Z = −2.03	**0.02**
Body mass 1^st^ infection	12	20.54±0.47	12	20.48±0.29	t = −0.18	0.86
Body mass 6^th^ infection^1^	12	22.65±0.53	12	22.86±0.53	F = 0.08	0.78
Body mass at dissection	7	21.69±1.68	7	22.49±0.40	t = 0.48	0.65
**Females**						
Spleen mass	10	0.09±0.01	10	0.06±0.00	Z = −1.83	**0.03**
Liver mass	10	1.13±0.08	10	1.04±0.06	Z = −1.68	**0.05**
Body mass 1^st^ infection	12	19.50±0.60	12	18.11±0.66	t = −1.67	0.12
Body mass 6^th^ infection^2^	10	20.10±0.47	10	19.89±0.47	F = 0.11	0.75
Body mass at dissection^3^	10	20.13±0.58	10	19.71±0.58	F = 0.24	0.63

The test values presented are Z from Wilcoxon Signed Ranks test, t from paired samples t-test, and F from ANCOVA.

Note; covariates:

1 Body mass prior to 1^st^ infection as a covariate in ANCOVA F = 7.75, P = 0.01

2 Body mass prior to 1^st^ infection as a covariate in ANCOVA F = 23.33, P<0.001

3 Body mass prior to 1^st^ infection as a covariate in ANCOVA F = 14.71, P = 0.001

**Table 2:** Pair-wise brother-sister comparisons for experimentally infected mice for mortality, *Salmonella* prevalence, *Salmonella* load and spleen and liver mass.

	Brothers	Sisters	Test	P
Mortality	36 % (4 / 11)	18 % (2 / 11)	Fisher's	0.64
*Salmonella* prevalence	100 % (6 / 6)	50 % (3 / 6)	Fisher's	0.18
*Salmonella* load	2.35±0.51 (6)	1.45±0.75 (6)	t = −1.00	0.37
Spleen mass	1.17±0.86 (6)	−0.02±0.22 (6)	Z = −1.15	0.25
Liver mass	1.43±0.57 (6)	−0.38±0.23 (6)	Z = −2.20	**0.03**

Differences in mortality and *Salmonella* prevalence were tested with Fisher's exact tests, *Salmonella* load (log_10_ colony forming units / ml) with paired samples t-test, and spleen and liver mass (residuals from a linear regression of organ mass on body mass) with Wilcoxon Signed Ranks test. Sample sizes are given in parenthesis.

**Figure 1:**
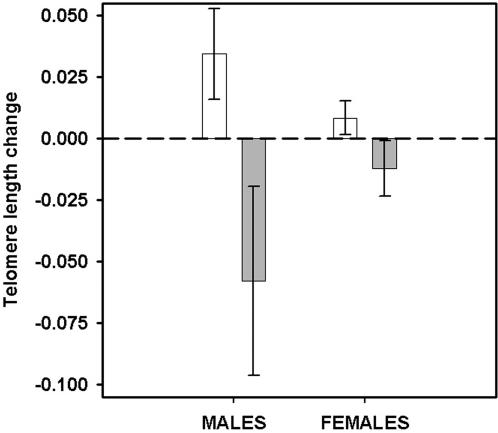
The consequences of infection on telomere length. Change of telomere length (mean±s.e.m.) of WBCs from sham-infected controls (white bars) versus infected mice that still harboured *Salmonella* at termination (grey bars). Negative values indicate telomere attrition and positive values telomeric gain.

We examined changes in telomeres in WBCs over nine months and five consecutive infections and found that the experimentally infected males showed significantly greater telomere attrition compared to sham-infected controls (Wilcoxon Signed Ranks test: Z = −2.35, *N* = 24, *P* = 0.01; the infected brother showed more attrition compared to sham-infected brother in 10 out of 12 pair-wise sib-sib comparisons). Unlike males, infection did not affect the telomeres of the females (Z = −0.76, *N* = 20, *P* = 0.28; the infected sister showed more telomere attrition than sham sister in 7 out of 10 pair-wise comparisons), and this sex-difference in telomere dynamics could be due to males being more susceptible and incurring greater pathology from *Salmonella* infection than females (see above). When we excluded individuals that were resistant and cleared infection by the end of the experiment from our analyses, we found that infected males still had significantly shorter telomeres than controls (Z = −2.22, *N* = 22, *P* = 0.02; Fig. 1; the infected brother showed more attrition compared to sham in 9 out of 11 pair-wise comparisons) and infected females also showed a marginally significant difference from controls (Z = −1.75, *N* = 10, *P* = 0.05; Fig. 1; the infected sister showed more attrition than sham in 4 out of 5 pair-wise comparisons). The differences we found between the treatment and control mice were partly due to telomere shortening in infected animals, and, somewhat surprisingly, telomere elongation in sham-controls (which we have observed previously [5]). Taken together, these results provide experimental evidence for the idea that infection causes damage to telomeric DNA, and are consistent with observational findings that lymphocytes develop shorter telomeres due to repeated antigenic stimulation [3], [4], [9]. An alternative possibility we can not rule out is that infection causes a shift in lymphocyte cell subpopulations having shorter telomeres [10]. In this case, telomere length would nevertheless provide a biomarker of exposure to infectious agents.

We also examined telomere lengths of hepatocytes and splenocytes at termination of the experiment, and found that telomere lengths in these two tissue types were highly correlated (Pearson r = 0.69, *N* = 41, *P*<0.001), and did not differ from each other (paired samples t-test, t = −0.38, *N* = 41, *P* = 0.71). Contrary to our expectation, however, infection did not reduce telomere lengths of hepatocytes (ANOVA, treatment F = 0.09, *N* = 41, *P* = 0.48; sex F = 4.41, *P* = 0.04, treatment x sex interaction F = 0.10, *P* = 0.76) or splenocytes (ANOVA, treatment F = 0.20, *N* = 41, *P* = 0.41; sex F = 0.13, *P* = 0.73; treatment x sex interaction F = 0.01, *P* = 0.93). Treatment effects remained non-significant when excluding the individuals that cleared infection from the analyses (data not shown). There are several reasons which could explain our failure to find treatment effects on tissue telomere lengths. First, it is possible that telomeres in hepatocytes and splenocytes are not as susceptible to ROS and replicative senescence as WBCs. Second, we had less power to detect a difference in these tissues than WBCs, because we could only collect liver and spleen samples at a single time point, after dissection, and hence could not test the within subject change, which controls for the large individual variability in telomere lengths. Third, we failed to obtain spleen and liver samples from individuals that died, which omits from our analyses the mice most strongly impacted by *Salmonella*. Lastly, once it becomes systemic, *Salmonella* resides highly localized inside the phagocytes within lesions [29], and our whole-organ approach may have been insufficient to detect more localized effects within these organs.

**Figure 2:**
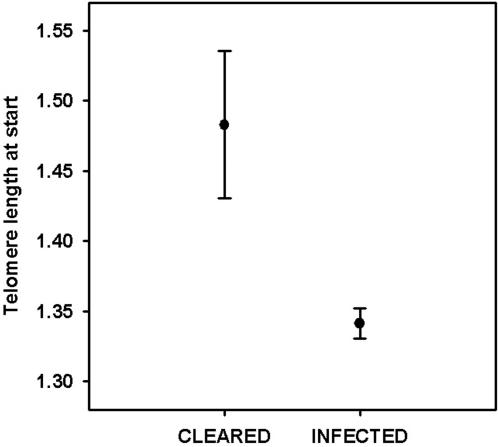
Initial telomere length and ability to resolve infection. WBC telomere lengths (mean T/S ratio±s.e.m.) at the beginning of the experiment for mice that cleared the infection (*N* = 6) versus mice that still harboured *Salmonella* (*N* = 11) at termination.

**Figure 3:**
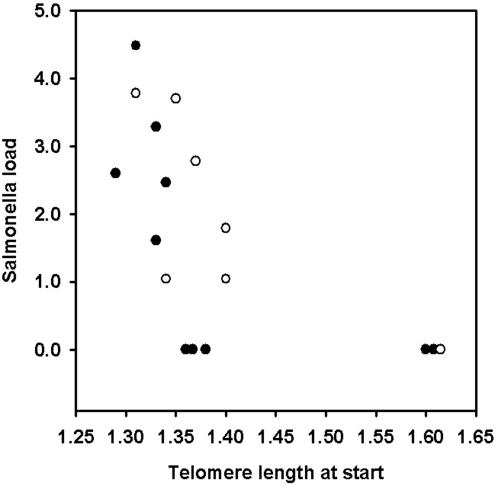
Initial telomere length and Salmonella load at termination. Relative telomere lengths (T/S ratio) at the start in WBCs from females (indicated with filled circles; *N* = 10) and males (open circles; *N* = 7) predicted *Salmonella* loads (log_10_ colony forming units/ml of spleen) at the end of the experiment.

Interestingly, we also found evidence that telomere length influences susceptibility to infectious disease. The mice that cleared *Salmonella* by the termination of the experiment, and hence were most resistant, had significantly longer WBC telomeres at the beginning than those that were still infected (Mann-Whitney U-test, U = 8.00, *N* = 17, *P* = 0.008; Fig. 2). Furthermore, individuals with relatively long telomeres at the beginning of the experiment had lower bacterial loads at termination (Spearman rank correlation, r_s_ = −0.72, *N* = 17, *P* = 0.0006; Fig. 3). These results suggest that individuals with long WBC telomeres at early age are more resistant to *Salmonella*, perhaps due to a higher proliferation capacity of leukocytes that increases efficiency of fighting infection [30], or perhaps telomere length reflects a more general aspect of individual quality [31] or ability to cope with stress [5], [6].

Finally, we examined the influence of telomere attrition rate on mortality, and found that the experimentally infected males that died during the last two infections showed a non-significant tendency for higher WBC telomere attrition rate during the first five infections compared to those that survived (−0.13±0.09 and −0.02±0.03, Mann-Whitney U-test, U = 8.00, *N* = 11, *P* = 0.16). This trend is consistent with observational findings in humans [4], [16], [32] and suggests that rapid telomere attrition could reduce ability to cope with infectious diseases during later life. A larger sample size is needed, however, to adequately test whether rapid telomere attrition reduces longevity.

A variety of mechanisms are suspected to contribute to senescence, and an important one is thought to be cumulative damage to telomeric DNA as a result to exposure to pathogens, inflammation and oxidative stress [1]–[4]. Our results provide experimental evidence for the idea that exposure to infectious diseases causes telomere attrition, and suggest that the variation in telomere length among individuals might be due, at least partly, to exposure to infectious diseases. Furthermore, our results support the idea that telomeres provide a biomarker for life-time exposure and ability to cope with inflammatory infections. Additional studies are needed to address whether pathogen-driven telomere attrition contributes to organismal senescence or otherwise reduces host fitness, and determine how pathogen-induced effects on telomeres interact with effects from other stressors, such as social and reproductive stress [5], [6].

## Materials and Methods

### Experimental animals

We used the F2 offspring of wild-caught house mice from Vienna, Austria. The mice were singly-housed in standard (type IIL) mouse cages under standard colony conditions, and provided laboratory rodent food (Altromin rodent diet 1324) and water *ad libitum*.

### Experimental infections and tissue sampling

We bred F1 offspring of wild-caught mice to produce 12 litters that had at least two female and two male offspring. We systematically assigned the animals either to treatment or control group. To control for potential familial effects, one sister-brother pair from each of the 12 litters was assigned to treatment group (12 males and 12 females) and another one to sham control group (12 males and 12 females). To obtain DNA for measuring telomeres in WBC, we collected 75 μl of blood from the tail vein of each mouse at the age of three months, and stored the blood in equal amounts of 50 mM EDTA at −80° C for DNA extraction. At the age of seven months, the treatment mice were orally infected with *Salmonella enterica* serovar Typhimurium, strain aroA (30 μl; 10^7^ colony forming units/ml). Thereafter, we re-infected mice repeatedly in four-week intervals. During each subsequent infection we added a novel strain to the inoculum (*S. enterica* serovar Enteriditis, strain 3b; *S.enterica* serovar Typhimurium, strain M525, LT2 and Sri-11; respectively), keeping the volume and dosage constant. The mice in the control group were sham-infected with the same volume of saline. After five subsequent infections, we collected blood from the mice (age 12 months) as previously. Immediately after the second bleeding, we infected the mice with the mixture of five different *Salmonella* strains, and re-infected them again with the same mixture four-weeks later. The rationale for using repeated inoculations with mixed strains is that we wanted to simulate more natural conditions under which individuals are faced with overlapping epidemics, where novel pathogen strains emerge as a result of mutation or invasion. The mice were restricted from food and water four hours prior to each inoculation to reduce variation in systemic invasion due to food in the gut. Two-weeks after the last infection, we euthanized the mice with CO_2_. We dissected the spleens and livers, and assessed *Salmonella* loads by counting the number of colony forming units/ml of spleen homogenates on selective agar plates [33]. The livers were snap-frozen and both the spleen and liver samples were stored at −80° C for DNA extraction.

### Telomere length measurement with real-time PCR

We extracted DNA using a commercial kit (DNeasy Blood and Tissue Kit, Qiagen) and measured the mean telomere lengths in WBC, splenocytes and hepatocytes using a real-time PCR method [28] we adapted for wild mice [5]. The procedure was carried out on an ABI (Applied Biosystems International) 7300 real-time PCR machine with SDS software, version 1.3.1 (ABI). This technique allows one to measure relative telomere length as a ratio of telomeric (T) DNA to a single-copy gene (S) DNA (T/S ratio). A specially designed oligonucleotide primer set hybridizes to the TTAGGG and CCCTAA repeats and selectively amplifies T DNA: longer telomeres lead to quantifiable acceleration of amplification. The quantity of T DNA is then divided by the quantity of S DNA of the same sample. We used *Mapk1* as the single-copy gene with the following primer sequences: *Mapk1*-F: 5′-GCTTATGATAATCTCAACAAAGTTCG-3′ and *Mapk1*-R: 5′-GATGTTCTCATGTCTGAAGCG-3′. Telomere length measurements using real-time PCR assume that quantitative differences in amplification products (T/S ratios) reflect variation in telomere length. This assumption is reasonable as several studies have previously confirmed that T/S ratios measured with real-time PCR are highly correlated with relative telomere restriction fragment (TRF) lengths [28], [34], [35]. Wells containing reference whole-mouse DNA (strain C57BL/6J, Jackson Laboratory) diluted over 16-fold range for T PCR (10, 5, 2.5, 1.25 and 0.625 ng per well) and for S PCR (5, 2.5, 1.25, 0.625 and 0.312 ng per well) were included in each PCR run so that the quantity of targeted templates in each research sample could be determined relative to the reference DNA sample by the standard curve method. In our analyses, no template controls (NTC) and standard curves were run with sample DNAs, all in quadruplicates in each PCR. The WBC DNA samples from the first bleeding and the second bleeding were run on adjacent wells, and the DNA samples from infected mice were run on same plates with those from sham-infected sibling controls. The final reaction wells for both T and S PCR contained 10 μl of ABI Syber Green Master Mix, 300 nM of forward and 300 nM of reverse primer, ≈5 ng of sample DNA, and enough double-distilled water to yield a 20 μl reaction. The thermal cycle conditions for T PCR were set at 94° C for 10 min, followed by 40 cycles at 95° C for 15 s, 56° C for 1 min and 68° C for 30 s with signal acquisition, and for the S PCR at 94° C for 10 min, followed by 40 cycles at 95° C for 15 s, 54° C for 20 s, and 72° C for 30 s with signal acquisition.

### Statistical analyses

Before conducting statistical tests (SPSS 15.0), we examined whether the data were normally distributed and had equal variances using Shapiro-Wilk and Levene's test. Whenever the data did not meet the criteria for parametric tests, we used non-parametric tests. We had *a priori* predictions for the direction of telomere attrition rate in WBC (treatment > sham control), telomere lengths in spleen and liver (treatment < sham control), for the effects of initial WBC telomere length on susceptibility to infection (short telomeres – low resistance), and telomere attrition rate on mortality risk (high attrition – increased mortality), and therefore, we used “directed tests”, rather than one- or two-tailed tests [36]. However, because we have confirmed other reports that *Salmonella* infection causes enlarged spleens and livers [33], we used one-tailed tests to compare differences in the organs of infected mice versus sham controls. We used residuals from a linear regression of spleen and liver mass on body weight when testing the differences in between experimentally infected brothers and sisters to control for differences in organ mass due to higher body masses in males. The α-level for statistical significance was set at 0.05. The averages are given as mean±s.e.m. The sample sizes differed among experimental groups because two of the treatment females died before the second bleeding, and five of the treatment males died during the last two infections. One of the males died because of a water-bottle accident, and was therefore excluded from mortality analyses.
